# Abandon of intramuscular administration of rabies immunoglobulin for post-exposure prophylaxis in the revised guidelines in the Netherlands in 2018: cost and volume savings

**DOI:** 10.2807/1560-7917.ES.2020.25.38.2000018

**Published:** 2020-09-24

**Authors:** Imke Schreuder, Cornelis De Pijper, Rob van Kessel, Leo Visser, Hans van den Kerkhof, on behalf of the Dutch advisory committee on rabies

**Affiliations:** 1 National Institute for Public Health and the Environment (RIVM), Bilthoven, the Netherlands; 2 Dutch advisory committee on rabies; further members of the network are acknowledged at the end of the article; 3 Centre for Tropical Medicine and Travel Medicine, Amsterdam UMC, University of Amsterdam, Amsterdam, the Netherlands; 4 Municipal Health Service Utrecht region, Zeist, the Netherlands; 5 Department of Infectious Diseases, Leiden University Medical Center, Leiden, the Netherlands

**Keywords:** rabies, immunoglobulin, post exposure prophylaxis, indication, guideline, the Netherlands

## Abstract

The World Health Organization (WHO) issued an updated position paper on rabies in 2018, mainly focusing on simplification of vaccination schedules and use of rabies immunoglobulin (RIG). The maximum amount of RIG anatomically feasible should be infiltrated exclusively in and around the wound and will no longer be calculated solely based on body weight. We describe the practical guideline implementing the revised RIG policy in the Netherlands on how to determine the amount of RIG for local administration. We calculated savings achieved through the revised WHO policy. We used information from a national database including rabies consultations in the Netherlands and clinical information from a public health service, clinical practitioners and national data on the amount of distributed RIG. Between 2008 and 2019, 5,164 consultations were registered. The most frequently affected anatomical location was hand or leg (43%). Around 80% concerned minor injuries (< 2 cm). From January 2016 to end December 2019, 7,361 mL RIG were distributed for 1,042 possible rabies exposures (EUR 1.4 million). Since implementing the revised policy, the amount of RIG distributed per order has sharply decreased (59%). Infiltrating RIG only locally saved large quantities of human RIG (EUR 1.1 million during 4 years) in the Netherlands.

## Background

Rabies is a lethal zoonotic viral disease responsible for an estimated 59,000 human deaths every year. So far, there is no effective treatment regimen and rabies is almost invariably fatal once clinical signs appear [1,2]. Most cases occur in Africa and Asia, with ca 40% of cases in children younger than 15 years [3,4]. While all mammals are susceptible to infection by rabies virus (RABV), dogs are able to transmit the virus more easily and are responsible for up to 99% of human rabies cases in rabies-endemic regions [2]. In the Netherlands, rabies is a rare disease with only four imported cases among travellers in the past 50 years [5,6]. Besides travellers, domestic exposure occurs occasionally through illegally imported dogs from rabies-endemic areas or through contact with bats that may carry European bat lyssaviruses [7,8].

## World Health Organization recommendations

The World Health Organization (WHO) issued an updated position paper in April 2018, mainly focusing on adjustments in pre-exposure prophylaxis (PrEP) and post-exposure prophylaxis (PEP) [9]. Prevention of rabies relies on several strategies such as the awareness of transmission risks from potentially rabies-infected animals and administering pre-exposure vaccination. After suspected or proven exposure to RABV, PEP consists of several steps including prompt washing of the wound, a series of vaccinations and administering rabies immunoglobulin (RIG) at the wound site up to the maximum calculated volume for category III exposures in previously unvaccinated individuals [2,9,10]. In particular, RIG is very costly to produce and in short supply worldwide resulting in the inability to adequately immunise exposed persons in many countries.

A shortcoming of the WHO update is that practical guidelines on the implementation of the revised RIG policy have not been provided. As the maximum amount of RIG will no longer be calculated solely on basis of body weight, determining the optimal amount of RIG that should be infiltrated in the wound strongly depends on the assessment by the clinical practitioner, based on nature and size of the injury.

## Situation in the Netherlands

In the Netherlands, RIG of human origin (HRIG) is used and only available from a national stock at the National Institute for Public Health and Environment (RIVM). Equine rabies immunoglobulin (ERIG) is not used in the Netherlands [11]. Before release, professional consultation with specialists working at the RIVM is compulsory since HRIG is not officially registered as a medication in the Netherlands. If a doctor orders more HRIG than practically can be administered locally, residual HRIG cannot be easily returned and must therefore be discarded. In 2019, the price of HRIG in the Netherlands was EUR 187.27 per mL (excluding accompanying costs such as transport and consultation with a pharmacist/doctor).

Shortly after WHO`s update in April 2018, a Dutch advisory committee was convened to discuss the revised policy for PrEP and PEP and its implications for the Dutch rabies immunisation guidelines. Most recommendations regarding PEP after a possible exposure were subsequently included in the guideline on rabies of the RIVM [11]. The updated Dutch guidelines for PrEP developed by the National Coordination Centre for Traveller Advice are in line with these recommendations.

The update has led to some important changes for PEP, including changes in the administration of RIG to patients with category III exposures [11]. RIG should be administered exclusively at the wound site in order to optimally neutralise RABV locally before the vaccine has induced a systemic immune response. Infiltration of an adequate dose of RIG in the wound area is effective [12]. However, the benefit from additional IM administration of the remaining RIG at a site distant to the wound is likely to be very limited [2,9]. Therefore, the advisory committee followed WHO`s recommendation that RIG should no longer be administered IM and started the new policy in November 2018 [11].

In this report, we aim to provide a practical guideline on how to assess the amount of HRIG for several injuries. In addition, we calculated the impact of the revised policy on the total amount of HRIG prescribed for exposed persons. We also assessed potential financial savings on a national scale.

## Methods and sources

We used a number of sources to meet our study goals:

- Background data from a unique online database containing all consultations on infectious diseases at the RIVM from 1 January 2008 to 31 December 2018 was used to assess the administered quantities of HRIG per year and type of injury for which they were indicated. These consultations included animal-associated incidents (AAI) with possible rabies exposure, both domestic and travel-related.
- Clinical information for 2018 was obtained from the Public Health Service (PHS) of The Hague in the Netherlands. This PHS registered all persons for whom HRIG was indicated. The data included basic information about the incident (personal details, country, animal etc.), medical information about the anatomical location and type of injury, the amount of HRIG that was infiltrated locally and the amount of HRIG left for IM administration.
- A small telephone survey was done in 2018 among 30 clinical practitioners in the Netherlands, to learn more about the cause and type of injuries they encountered in daily practice for which HRIG was indicated. In this inventory, injuries were described in more detail and subsequently categorised by size (minor was defined as ≤ 2 cm, large as > 2 cm).
- National information from the RIVM on HRIG distribution from 1 January 2016 to 31 December 2019 was used to compare the amounts distributed before and after the introduction of the revised policy.

For the calculation of changes in the use of HRIG and related savings, we combined outcomes of all four sources. In addition, we searched PubMed using the Medical Subject Headings (MeSH) terms ‘rabies immunoglobulin’ AND ‘local administration’. We included only articles in English published between 1 January 2009 and 1 September 2019 which reported on local administration of HRIG or ERIG.

## Findings

### Information on national rabies consultations

In total 5,164 consultations for AAI with possible rabies exposure were registered between 1 January 2008 and 31 December 2018 in the national web-based tool. After review, 3,143 of these concerned category III exposures. Of those, 1,091 persons (35%) had an indication for HRIG, the others did not, owing to factors such as vaccination started > 7 days before or low-risk incidents involving for example monkeys. Overall, dogs (45%; n = 1,423), cats (19%; n = 597), monkeys (16%; n = 505) and bats (11%; n = 336) were most frequently implicated in category II events, mainly causing injuries in hands, fingers or wrists (36%; n = 1,126). The number of consultations for which HRIG was possibly indicated increased almost two-fold from 2013 (n = 235) to 2018 (n = 438).

### Amount of human rabies immunoglobulin per wound type, savings and costs

The small inventory among 30 clinical practitioners in the Netherlands in 2018 showed that an estimated 80% of injuries from with a category III exposure concerned minor injuries (≤ 2 cm), for which less than 2 mL of HRIG would be sufficient to administer locally. Most injuries among travellers involved incidents with dogs. In addition, the 35 type III exposures recorded by the PHS the Hague were linked to incidents with dogs (n = 20), cats (n = 10) or bats (n = 5). The anatomical locations concerned most frequently were hand or leg (both n = 15). After bat incidents, an average maximum of 1.5 mL HRIG could have been infiltrated locally in fingers or toes. This is only around 20% of the average total volume of HRIG ordered for an IM administration based on body weight (7.4 mL). For incidents involving a dog or cat, these percentages were 50% (5.6/11.1 mL) and 40% (3.7/9.2 mL) of the average ordered volume, respectively.

In addition, national data on HRIG distribution from the RIVM from 1 January 2016 to 31 December 2019 showed that a total of 7,361 mL HRIG was distributed for 1,042 HRIG orders, resulting in a total cost of HRIG for these years of EUR 1.4 million (Table 1). The average amount of HRIG ordered per patient based on body weight from 2016 to 2018 was 9.4 mL. Since the implementation of the revised HRIG policy in November 2018, the amount of HRIG distributed per order has sharply decreased to an average of 3,81 mL HRIG per order in 2019 (Table 1). This is a decrease of 59% compared with the average 9.4 mL in the years before the policy change in November 2018, resulting in an expenditure of EUR 1,047 per order, which was EUR 1,1 million over the 4-year period in the Netherlands, excluding accompanying costs. However, despite the decrease in HRIG volume, the number of orders increased considerably over time (Figure) from 202 in 2016 to 383 in 2019).

**Table 1:** Human rabies immunoglobulin distributed in the Netherlands, 1 January 2016–31 December 2019 (n = 1,042 orders)

Year	HRIG (mL)	HRIG orders (n)	Average volume HRIG/person (mL)
2016	1,979	202	9.85
2017	1,873	195	9.70
2018^a^	2,054	262	8.12
2019	1,455	383	3.81
**Total**	**7,361**	**1,042**	**7.87**

HRIG: human rabies immunoglobulin.

^a^ Until 1 November 2018, HRIG was based solely on body weight.

**Figure:**
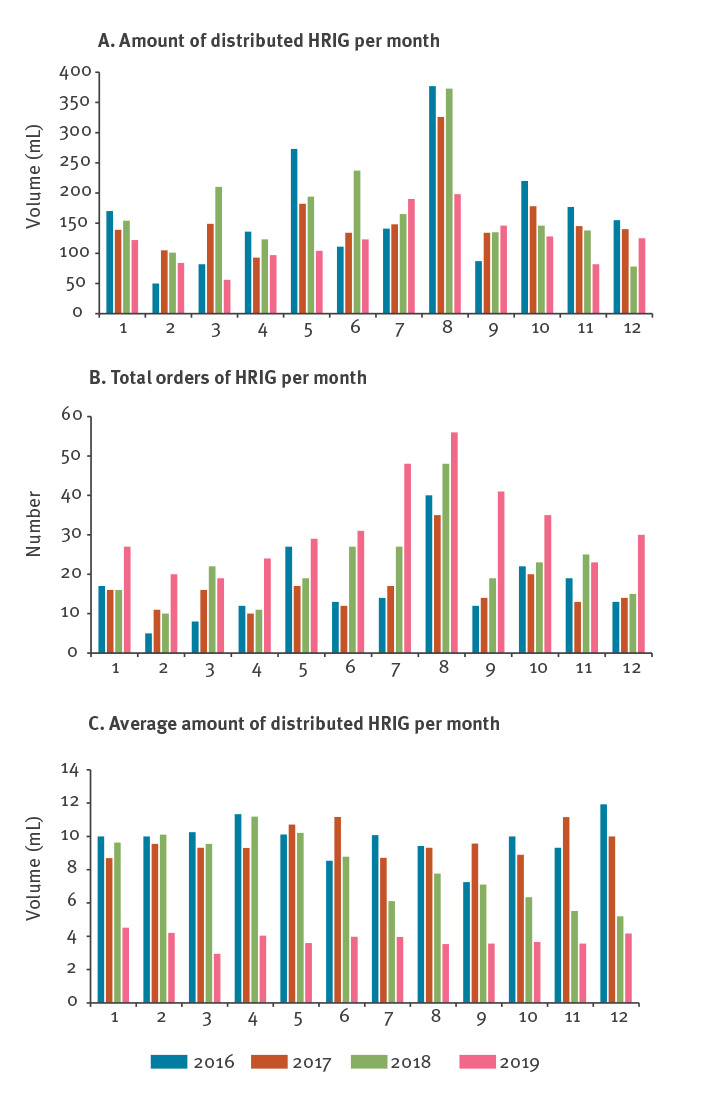
Extradition of human rabies immunoglobulin in the Netherlands, 1 January 2016–31 December 2019

HRIG: human rabies immunoglobulin.

### Literature search

Based on the MeSH terms and timeline used in our brief literature search, 41 studies were published on these topics but only two studies by Barthi et al. in India were suitable for our analysis [13,14]. In the references of these studies and using the option ‘similar articles’ in PubMed, no other useful studies emerged. Barthi et al. showed that local infiltration of RIG without systemic IM administration was not associated with a higher rabies mortality and was a cost-effective approach for passive immunisation against rabies, reducing the costs as much as 88%. Besides the availability of studies, no other practical guidelines on the implementation of the revised RIG policy appear to be provided internationally, apart from Belgium which to a great extent followed the Dutch guideline (Table 2) [15]. The Netherlands may have adapted early to the WHO`s revised policy.

**Table 2:** Practical guideline of the indication of the amount of HRIG for local infiltration in and around the wound related to anatomical location of the injury, the Netherlands [11]

Anatomical location	Minimum volume to be ordered per wound^a^	Maximum volume to be ordered^b^
Finger/toe	2 mL	2 mL
Hand/foot	2 mL	4 mL
Knee/ankle/wrist/elbow	2 mL	6 mL
Forearm/lower leg	2–4 mL	10 mL
Upper arm/thigh/trunk	4 mL	10 mL
Face/scalp	2 mL	10 mL
Mucosal contact without injury	No HRIG	No HRIG

ERIG: Equine rabies immunoglobulin; HRIG: human rabies immunoglobulin; IU: international unit.

^a^ Note that the volume of HRIG to be ordered may exceed the maximum volume based on body weight. This especially applies to children (and some adults with low body weight). Different calculations apply for ERIG.

^b^ Maximum based on body weight (20 IU/kg), to be calculated with the formula: (body weight in kg × 20) / 150 IU = maximum allowed mL HRIG. Different calculations apply for ERIG.

For additional information see the national guideline on rabies (in Dutch) [11].

### Practical guideline

Based on all sources described above, a practical national guideline on the implementation of the revised RIG policy was formulated as shown in Table 2. Thereafter, the guideline was reviewed by the members of the Dutch advisory committee on rabies. In addition to these members, several clinical practitioners were asked to provide their feedback on the proposed guideline. As illustrated in Table 2, it provides indications of the minimum and maximum amount of HRIG that is expected to be required for infiltration in the wound for several anatomical locations. The practical guideline only applies to the calculation of HRIG (different calculations apply for ERIG). It has been published as an annex to the National Dutch guideline on rabies [11]. Since the implementation of the revised RIG policy in November 2018, HRIG has been applied successfully because none of the people with AAI developed disease in the Netherlands and no major adjustments were needed.

## Discussion

After recent adjustments to the WHO rabies policy, the Dutch Centre for Infectious Diseases Control has provided a practical guideline on how to assess the amount of HRIG for several injuries when administered only locally to the wound. The practical guideline is based on different data sources for which consensus was reached among experts of the Dutch advisory committee on rabies. Our results show that since the implementation of the revised HRIG policy in the Netherlands in November 2018, the amount of HRIG in mL distributed per order has decreased sharply. Therefore, the total distributed amount of HRIG has decreased although the number of orders has risen considerably. This may be largely attributable to changed travel behaviour in the Dutch population as well as increased awareness of rabies infection risks. 

Our results show that local infiltration of RIG without systemic IM administration has led to a considerable reduction in the amount of HRIG used in the Netherlands after implementing the revised HRIG policy and a reduction in accompanying costs for passive immunisation to prevent rabies infection after possible exposure. Especially for low- and middle-income countries, but also high-income countries, administering RIG only at the wound site makes PEP accessible for more patients with the same budget. Even more important is the reduction because RIG is a product that is in very short supply worldwide and that should be reserved for high-risk indications, mostly in low-income countries endemic for rabies. The use of RIG can further be avoided by emphasising PrEP in people who are at increased risk of rabies infection.

Although our study can contribute to nationwide policies in other countries that are planning to implement the revised policy for HRIG administration, the results should be interpreted in the context of some limitations. Firstly, the study was originally not designed specifically to compare HRIG administration before and after the implementation of the revised policy. Preferably, such a study would have to be conducted in several multi-country settings. However, the Netherlands adapted to the WHO`s revised policy in November 2018, with positive outcomes in terms of feasibility and costs [15]. If all countries implemented the revised policy, we would all contribute to saving RIG, an expensive and scarce product for rabies-endemic countries who need it the most. Secondly, the amount of HRIG saved per person was calculated for a short period. However, these first results showed a sharp decrease in the volume of HRIG used. Thirdly, the exact amount of HRIG depends on the assessment by the treating clinician on the basis of pictures or inspection on site. The amount of ordered HRIG per case may therefore vary. Fourthly, we did not perform a systematic review on the practice of RIG administration. To the best of our knowledge after searching PubMed, however, no studies on this practice have been conducted recently and as far as we know, no other practical guidelines on the implementation of the revised RIG policy had been developed internationally by summer 2019, except in Belgium. Belgian public health colleagues published a practical guideline in May 2019, which included the Dutch recommendations on RIG administration [15]. Since then, however, the recommendations of some other European countries have been revised such as the guidelines on rabies PEP in Denmark and Sweden. Finally, the small survey indicated that an estimated 80% of injuries among people with a category III exposure may concern minor injuries. This is probably due to the relatively large number of incidents with bats in the Netherlands (± 70 incidents/year), the only mammals in our country that transmit lyssaviruses.

## Conclusions

We provide a practical guideline on how to assess the amount of HRIG for several injuries, which may help other countries implement the revised policy of RIG administration as proposed by the WHO in 2018. The revised policy on the total amount of RIG prescribed for exposed individuals has led to a considerable reduction in the costs associated with passive immunisation against rabies infection. This makes a full PEP for high-risk exposures more affordable and accessible, which is of particular relevance in low and middle-income countries where RABV is more often endemic and contributes to the goal of zero human rabies deaths by 2030 worldwide [2].

## References

[1] Fooks AR Banyard AC Horton DL Johnson N McElhinney LM Jackson AC Current status of rabies and prospects for elimination. Lancet. 2014;384(9951):1389-99. 10.1016/S0140-6736(13)62707-5 24828901 PMC7159301

[2] World Health Organization (WHO). WHO expert consultation on rabies – third report. Geneva: WHO; 2018. Available from: https://apps.who.int/iris/handle/10665/272364

[3] Singh R Singh KP Cherian S Saminathan M Kapoor S Manjunatha Reddy GB Rabies - epidemiology, pathogenesis, public health concerns and advances in diagnosis and control: a comprehensive review. Vet Q. 2017;37(1):212-51. 10.1080/01652176.2017.1343516 28643547

[4] Hampson K Coudeville L Lembo T Sambo M Kieffer A Attlan M Estimating the global burden of endemic canine rabies. PLoS Negl Trop Dis. 2015;9(4):e0003709. 10.1371/journal.pntd.0003709 25881058 PMC4400070

[5] Wieten RW Tawil S van Vugt M Goorhuis A Grobusch MP Risk of rabies exposure among travellers. Neth J Med. 2015;73(5):219-26. 26087801

[6] Carrara P Parola P Brouqui P Gautret P Imported human rabies cases worldwide, 1990-2012. PLoS Negl Trop Dis. 2013;7(5):e2209. 10.1371/journal.pntd.0002209 23658853 PMC3642086

[7] Shope RE Rabies-related viruses. Yale J Biol Med. 1982;55(3-4):271-5. 6758373 PMC2596466

[8] Schneider LG Antigenic variants of rabies virus. Comp Immunol Microbiol Infect Dis. 1982;5(1-3):101-7. 10.1016/0147-9571(82)90021-2 6181927

[9] World Health Organization Rabies vaccines: WHO position paper, April 2018 - Recommendations. Vaccine. 2018;36(37):5500-3. 10.1016/j.vaccine.2018.06.061 30107991

[10] O’Brien KL Nolan T SAGE WG on Rabies The WHO position on rabies immunization - 2018 updates. Vaccine. 2019;37(Suppl 1):A85-7. 10.1016/j.vaccine.2018.10.014 30342901 PMC6863036

[11] Centre for Infectious Diseases Control. Rabies guideline [in Dutch]. Bilthoven, The Netherlands: National Institute for Public Health and the Environment; 2018.

[12] Bharti OK Thakur B Rao R Wound-only injection of rabies immunoglobulin (RIG) saves lives and costs less than a dollar per patient by "pooling strategy". Vaccine. 2019;37(Suppl 1):A128-31. 10.1016/j.vaccine.2019.07.087 31395454

[13] Bharti OK Madhusudana SN Gaunta PL Belludi AY Local infiltration of rabies immunoglobulins without systemic intramuscular administration: An alternative cost effective approach for passive immunization against rabies. Hum Vaccin Immunother. 2016;12(3):837-42. 10.1080/21645515.2015.1085142 26317441 PMC4964710

[14] Bharti OK Madhusudana SN Wilde H Injecting rabies immunoglobulin (RIG) into wounds only: A significant saving of lives and costly RIG. Hum Vaccin Immunother. 2017;13(4):762-5. 10.1080/21645515.2016.1255834 28277089 PMC5404375

[15] Soentjens P, Declercq S. Prophylaxie post-exposition contre la rage. [Rabies post-exposure prophylaxis]. Antwerp: Institute of Tropical Medicine; 2019. p. 16. French. Available from: https://www.itg.be/Files/docs/Reisgeneeskunde/PEP_Rabies_FR.pdf

